# Prediction of prognosis in patients with severe COVID-19 pneumonia using CT score by emergency physicians: a single-center retrospective study

**DOI:** 10.1038/s41598-023-31312-5

**Published:** 2023-03-10

**Authors:** Yasufumi Oi, Fumihiro Ogawa, Tsuneo Yamashiro, Shoichiro Matsushita, Ayako Oguri, Shusuke Utada, Naho Misawa, Hiroshi Honzawa, Takeru Abe, Ichiro Takeuchi

**Affiliations:** 1 Emergency Care Department, Yokohama City University Hospital, 3-9 Fukuura, Kanazawa-ku, Yokohama, Kanagawa 236-0004 Japan; 2 Department of Emergency Medicine, Yokohama City University School of Medicine, Yokohama, Japan; 3 Department of Radiology, Yokohama City University School of Medicine, Yokohama, Japan; 4 Advanced Critical Care and Emergency Center, Yokohama City University Medical Center, Yokohama, Japan

**Keywords:** Anatomy, Health care

## Abstract

We aimed to develop a method to determine the CT score that can be easily obtained from CT images and examine its prognostic value for severe COVID pneumonia. Patients with COVID pneumonia who required ventilatory management by intubation were included. CT score was based on anatomical information in axial CT images and were divided into three sections of height from the apex to the bottom. The extent of pneumonia in each section was rated from 0 to 5 and summed. The primary outcome was the prediction of patients who died or were managed on extracorporeal membrane oxygenation (ECMO) based on the CT score at admission. Of the 71 patients included, 12 (16.9%) died or required ECMO management, and the CT score predicted death or ECMO management with ROC of 0.718 (0.561–0.875). The death or ECMO versus survival group (median [quartiles]) had a CT score of 17.75 (14.75–20) versus 13 (11–16.5), *p* = 0.017. In conclusion, a higher score on our generated CT score could predict the likelihood of death or ECMO management. A CT score at the time of admission allows for early preparation and transfer to a hospital that can manage patients who may need ECMO.

## Introduction

The World Health Organization (WHO) officially declared coronavirus disease 2019 (COVID-19) a pandemic on March 11, 2020, and currently (October 2022) there are more than 680 million confirmed cases and 6.5 million deaths from COVID 19 worldwide^1^. Although COVID-19 is a highly contagious disease, the mortality rate is relatively low (1–3.5%), except in elderly patients with multiple underlying medical conditions. However, severe pneumonia develops in 15–20% of those affected, and intensive care unit (ICU) treatment is required in 5–10%^2^.

Currently, vaccines and treatments against severe acute respiratory syndrome coronavirus 2 (SARS-CoV-2) are increasingly being developed worldwide, and although the number of COVID-19 patients is still high, these measures have reduced the severity of the disease and improved its prognosis. However, treatment depends on the severity of COVID-19 symptoms, and severe cases require admission to the ICU. Reverse transcriptase-polymerase chain reaction (RT-PCR) is the most reliable diagnostic method for COVID-19^3^, but computed tomography (CT) also has a high sensitivity for the diagnosis of COVID-19. The most common CT findings in patients with COVID-19 are ground-glass opacity (GGO) and consolidation^4,5^. In the early stages of COVID-19, clinical and imaging features are most important for establishing a diagnosis, assessing changes in severity, and adjusting treatment plans^6^.

Since the outbreak of this pandemic, COVID-19 has presented a pneumonia image on CT and an objective numerical CT score has been reported to indicate the extent to which the pneumonia image is occupied^7–9^. Most CT scores are read by a diagnostic radiologist, who anatomically divides the CT image into five lobes, three on the right and two on the left, and assigns a score for the extent of pneumonia to each lobe. The scoring obtained from these can be useful in predicting patient prognosis. However, one of the problems with this is that scoring from CT itself can be difficult and is not possible at every facility. In Japan, it is common for radiologists to read CT images taken and make a presumptive diagnosis on the CT images under the final check by a radiologist. However, non-radiologists such as primary care physicians, surgeons, and emergency physicians also have the skills to read CT images; our results show that the severity of illness did not differ when a skilled and experienced emergency physician read the images, without the need for a radiologist to perform a real-time, specialized reading. The CT score proposed in this study is relatively simple and easy to calculate and can be read by emergency physicians who are likely to see COVID patients first, not radiologists. In this study, the readings of emergency physicians and radiologists were also compared, and the prognostic prediction between the two was discussed. Thus, our CT score can provide physicians with clues about possible clinical outcomes without relying on radiologists, who are not readily available, especially in third-world countries. This study aimed to develop a CT score that can be used by non-radiologist physicians to predict prognosis and correlate it with clinical outcomes.

## Methods

This was a retrospective study conducted using the data of patients with COVID-19 (except for outpatients with mild COVID-19) who underwent standard treatment. All patients were intubated with intensive care at Yokohama City University Hospital from June 2020 to September 2021. This was a single-center, retrospective analysis. All patients had positive RT-PCR results and underwent a chest CT at the time of tracheal intubation. The current study compared two groups of patients: those who died or were placed on extracorporeal membrane oxygenation (ECMO) and those who survived only on ventilator management.

Age, sex, and weight were considered as individual-specific information. The time from onset to oxygen administration, from onset to hospitalization, from onset to tracheal intubation, and from onset to CT imaging, were considered. Additionally, the time from admission to CT imaging and from intubation to CT imaging were considered. Systolic blood pressure, heart rate, respiratory rate, body temperature, and SpO_2_ were considered as vital signs on admission. For ventilation-related information, we examined airway pressure at intubation, airway pressure at intubation > 26.5 cmH_2_O, maximum airway pressure during hospitalization, maximum positive end-expiratory pressure (PEEP) during hospitalization, and tidal volume during hospitalization. Medical history included the presence or absence of maintenance dialysis, presence or absence of hypertension, and presence or absence of diabetes mellitus. The Coronavirus Clinical Characterisation Consortium (4C) mortality score, Acute Physiology and Chronic Health Evaluation (APACHE II) score, Sequential Organ Failure Assessment (SOFA) score, and Simplified Acute Physiology Score (SAPS II) were evaluated for the prognostic score. For laboratory findings on admission, we examined the patients’ white blood cell (WBC) and platelet counts, and creatinine, bilirubin, blood urea nitrogen (BUN), and brain natriuretic peptide (BNP) levels. Also we examined their blood gas analysis on admission, including the PaO_2_/F_I_O_2_ (P/F) ratio, pH, and HCO3–. Other variables that were considered are as follows: maximum C-reactive protein (CRP) level during hospitalization, prone position during hospitalization, tracheostomy during hospitalization, and presence of bimodal inflammatory changes (i.e., CRP improves once and falls below 4, but the inflammatory response of CRP = 10 or higher persists) during hospitalization. The primary outcomes were death or ECMO management. The secondary outcome was the comparison of the prognostic value of CT scores between emergency physicians and radiologists. The study was approved by the Ethics Committee of Yokohama City University (B200200048) and all patients provided written informed consent. The research was performed in accordance with the Declaration of Helsinki.

### Clinical workflow and disease staging

All patients underwent routine blood tests and arterial blood gas (ABG) testing. Patients with pneumonia on CT and reservoir mask with oxygen 7 L/min or higher to maintain SpO_2_ 93% were intubated and ventilated. Patients who required ICU or ventilator management were classified as having severe COVID-19 in accordance with the guidelines of the Japanese Ministry of Health, Labour, and Welfare^10^. All COVID patients were treated with remdesivir (200 mg/day on day 1 and 100 mg/day on days 2 through 10) as an antiviral drug, dexamethasone 6.6 mg/day for 10 days as a steroid, and continuous heparin 10,000 U/day as an anticoagulant. Ventilator management was limited to a maximum PEEP of 15 cmH_2_O, and airway pressure did not exceed 30 cmH_2_O. The lung protection strategy aimed at a tidal volume of 6–8 mL/kg, and deep sedation and muscle relaxants were used if the patient presented with large excess breaths. If CT showed a strong image of pneumonia on the dorsal side and PaO_2_/F_I_O_2_ (P/F) was below 200, the patient was placed in the prone position. Venovenous extracorporeal membrane oxygenation (VV-ECMO) was introduced when oxygenation could not be maintained despite the above mentioned respiratory management. The indications for VV-ECMO were as follows: patients with hypoxemia with F_I_O_2_ ≥ 0.8 and P/F < 100, respiratory acidosis with pH ≤ 7.2 and plateau pressure > 32 cmH_2_O, Murray score > 3, or in the prone position despite treatment intervention for the original disease, lung protection strategy + high PEEP strategy and prone therapy, and poor response to therapy.

### CT score

**Figure 1:**
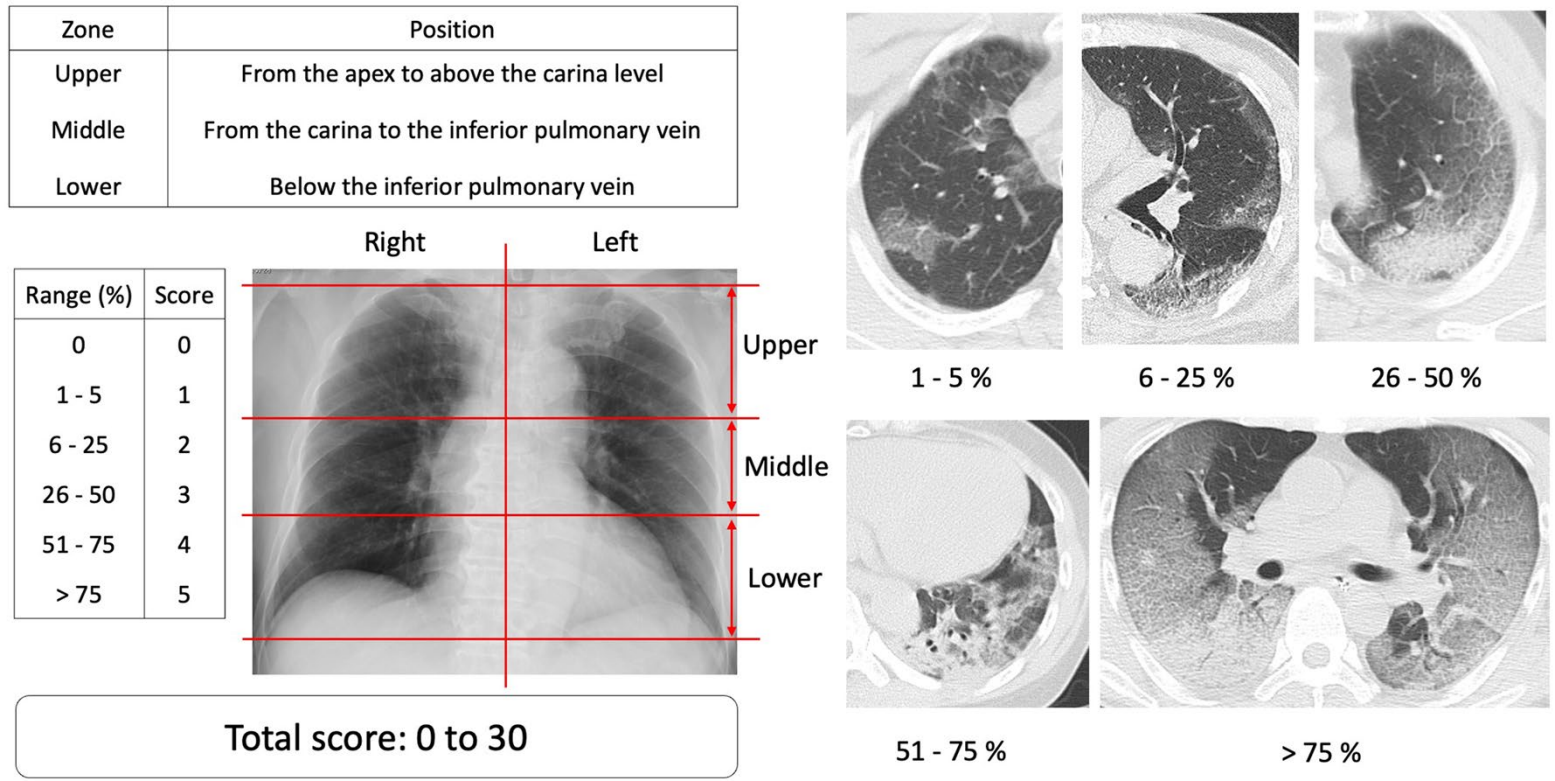
CT scores and axial images by lung zone involvement in COVID-19 pneumonia. The lung area was divided into three zones from the apex to bottom. The upper lung zone extended from the pulmonary apex to the bronchial bifurcation, the middle from the bronchial bifurcation to the right inferior pulmonary vein, and the lower from the right inferior pulmonary vein to the bottom.

Radiological terms such as GGO, crazy paving pattern, and consolidation were defined based on the definitions by the Fleischner Society for Chest Imaging^8^. In all cases, the CT was read using the axial mode only. The lung area was divided into three zones from the apex to bottom. The upper lung zone extended from the pulmonary apex to the bronchial bifurcation, the middle from the bronchial bifurcation to the right inferior pulmonary vein, and the lower from the right inferior pulmonary vein to the bottom. Thus, both lungs were separated to the six lung zones. Severity scores were calculated as follows, considering the extent of anatomical lesions: 0, no involvement; 1, less than 5% involvement; 2, 6–25% involvement; 3, 26–50% involvement; 4, 51–75% involvement; and 5, > 75% lesions. The resulting CT score was the sum of the scores of the six lung zones (the total score ranged 0–30) (Fig. 1).

To evaluate the level of agreement, we calculated Cronbach's Alpha between the following pairs: emergency physicians versus radiologists with all patients’ data; two emergency physicians with all patients’ data; two radiologists with all patients’ data; and two emergency physicians with mild to moderate COVID patients. A value ≥ 0.90 indicates excellent consistency and ≥ 0.80 indicates good consistency.

### Statistical analysis

Continuous variables were expressed as median (quartiles), and categorical variables as frequency (%). To compare two divided groups based on outcomes, Mann–Whitney test was used for a continuous variable and Fisher’s exact test for a categorical variable. Receiver operating curve (ROC) analysis was used to predict prognosis. To identify factors associated with the outcomes, we used a multiple logistic regression model with forward selection, controlling for CT score of 16.7 or higher, age, weight, and sex. The following factors were analyzed by forward selection: time from onset of illness to oxygen administration, time from onset of illness to tracheal intubation, presence of maintenance dialysis, presence of hypertension, presence of diabetes, maximal CRP during hospitalization, BNP on admission, presence of prone therapy, presence of tracheostomy, presence of bimodal inflammatory response, presence of GGO, presence of crazy paving pattern, presence of consolidation, 4C mortality score, APACHE II score, SOFA score, SAPS II, and presence of airway pressure of 26.5 cmH_2_O or higher at the time of intubation. Among these variables, 4C mortality score, airway pressure > 26.5 cmH_2_O at intubation and maximum CRP during hospitalization were selected. Statistical significance was set at *p* < 0.05. We used Stata 13 software (Stata Statistical Software: Release 13. College Station, TX: StataCorp LP) for the statistical analysis.

### Ethical approval and consent to participate

The study was approved by the Ethics Committee of Yokohama City University (B200200048). All the patients provided informed consent to participate in the study.

## Results

### Population, clinical presentation, and laboratory findings

Twelve patients (16.9%) died or were placed on ECMO and 59 (83%) survived on ventilator management alone. Age was 62.5 versus 61 years (*p* = 0.406) in the mortality or ECMO and survival groups, weight was 67.3 versus 72 kg (*p* = 0.890), and sex (male) was 75 versus 79.6% (*p* = 0.718), with no statistical difference between the two groups. As for the vital signs on admission, systolic blood pressure was 125 versus 135 (*p* = 0.228), heart rate was 75 versus 89 (*p* = 0.544), respiratory rate was 25 versus 25 (*p* = 0.993), body temperature was 37.5 versus 37.2 (*p* = 0.460), and SpO2 was 93.5 versus 94 (*p* = 0.847), with no statistical difference between the two groups. The time from disease onset to oxygen administration was 8 versus 6 days (*p* = 0.082), from onset to hospitalization, 7.5 versus 6 days (*p* = 0.089), from onset to tracheal intubation, 10 versus 8 days (*p* = 0.187), and from onset to CT scan, 7.5 versus 6 days (*p* = 0.119). Observations obtained from the ventilator showed that the airway pressure at intubation was 25.5 versus 23 cmH_2_O (*p* = 0.150) and ROC was 0.631 (0.438–0.824) for predicting death or ECMO group by airway pressure at intubation, with a cut-off value of 26.5, sensitivity of 50%, and specificity of 81%. The maximum airway pressure during the admission course was 29 versus 24 cmH_2_O (*p* < 0.0001), airway pressure higher than 26.5 cmH_2_O at intubation was 50 versus 18.6% (*p* = 0.020), the maximum PEEP required was 14.5 versus 12 cmH_2_O (*p* = 0.057), and the single ventilation rate was 6.47 versus 6.96 mL/kg (*p* = 0.673). There was no statistical difference in patients' history of pre-existing medical conditions: 8.3 versus 10.2% had maintenance dialysis (*p* = 0.846), 50 versus 42.4% had hypertension (*p* = 0.627), and 66.7 versus 42.4% had diabetes mellitus (*p* = 0.124). There was no statistical difference in prognostic scores: 12 versus 11 for the 4C mortality score (*p* = 0.110), 12 versus 10 for the APACHE II score (*p* = 0.239), 4 versus 4 for the SOFA score (0.225), and 33.5 versus 31 for SAPS (*p* = 0.213). For the laboratory findings on admission, WBC was 9300 versus 7600 (*p* = 0.240), platelet count was 21.15 versus 19.7 (*p* = 0.914), creatinine was 0.785 versus 0.75 (*p* = 0.213), bilirubin was 0.55 versus 0.5 (*p* = 0.703), BUN was 27 versus 20 (*p* = 0.113), and BNP was 21.8 versus 25.2 (*p* = 0.920), with no statistical difference between the two groups. Lastly, for the blood gas analysis on admission, P/F ratio was 158.4 versus 148.2 (*p* = 0.724), pH was 7.351 versus 7.436 (*p* < 0.0001), and HCO3- was 23.45 versus 24.6 (*p* = 0.184). Other factors were as follows: prone therapy was performed in 41.7 versus 22% (*p* = 0.152), bimodal inflammatory response was observed in 58.3 versus 42.4% (*p* = 0.311), tracheostomy was required in 25 versus 25.4% (*p* = 0.975), and the maximum CRP level during hospitalization was 24.3 versus 12.2 mg/dL (*p* = 0.012) (Table 1).

**Table 1:** Characteristics of patients at baseline.

	Death or ECMO		Survival group		*p*-value
	(n = 12)		(n = 59)		
Sex, no. (%)					
Men*	9	(75.0)	47	(79.6)	0.718
Age (yrs)	62.5	(57–75.5)	61	(52–70)	0.406
BW (kg)	67.3	(60.2–97.3)	72	(61.5–83)	0.890
From onset to					
Oxygen administration (day)	8	(6.5–9.5)	6	(5–9)	0.082
Hospitalization (day)	7.5	(6–9.5)	6	(4–8)	0.089
Intubation (day)	10	(7–12)	8	(6–11)	0.187
CT imaging (day)	7.5	(6–10)	6	(4–9)	0.119
From admission to CT imaging (day)	− 0.5	(-2.5–0)	0	(-1–0)	0.440
From intubation to CT imaging (day)	1	(0–3)	1	(0–2)	0.727
Ventilator settings					
Airway pressure at intubation (cmH_2_O)	25.5	(22–28)	23	(20–25)	0.150
Maximum airway pressure (cmH_2_O)	29	(27.5–30.5)	24	(21–25)	< 0.001
Airway pressure at intubation > 26.5cmH_2_O* (%)	6	(50.0)	11	(18.6)	0.020
Maximum PEEP (cmH_2_O)	14.5	(12–15)	12	(10–14)	0.057
Single tidal volume (mL/kg)	6.47	(5.33–8.20)	6.96	(5.64–7.63)	0.673
Complications					
Continuous dialysis* (%)	1	(8.3)	6	(10.2)	0.846
Hypertension* (%)	6	(50.0)	25	(42.4)	0.627
Diabetes mellitus* (%)	8	(66.7)	25	(42.4)	0.124
Prognostic score					
CT score	17.75	(14.75–20)	13	(11–16.5)	0.017
4C mortality score	12	(11–14.5)	11	(9–13)	0.110
APACHE II score	12	(10–13.5)	10	(8–14)	0.239
SOFA score	4	(4–7)	4	(3–6)	0.225
SAPS II score	33.5	(31.5–41)	31	(24–41)	0.213
Vital signs on admission					
Systolic blood pressure (mmHg)	125	(111.5–143)	135	(117–157)	0.228
Heart rate (beats/min)	75	(67–102.5)	89	(70–102)	0.544
Respiratory rate (breaths/min)	25	(24–29)	25	(20–30)	0.993
Body temperture (℃)	37.45	(36.35–37.9)	37.2	(36.6–38.3)	0.460
SpO_2_ (%)	93.5	(92–95)	94	(90–96)	0.847
Laboratory findings on admission					
White blood cell count (/μL)	9300	(6700–13,150)	7600	(5800–11,200)	0.240
Platelet count (10^3^/μL)	21.15	(15–24.7)	19.7	(15.3–26.1)	0.914
Creatinine (mg/dL)	0.785	(0.71–1.54)	0.75	(0.62–1.06)	0.213
Bilirubin (mg/dL)	0.55	(0.45–0.75)	0.5	(0.4–0.8)	0.703
Blood urea nitrogen (mg/dL)	27	(20–36.5)	20	(16–29)	0.113
BNP (pg/mL)	21.8	(8.4–61.45)	25.2	(8.1–50.3)	0.920
Blood gas analysis on admission					
P/F ratio	158.4	(92–189.5)	148.2	(94.9–207.7)	0.724
pH	7.351	(7.283–7.398)	7.436	(7.388–7.476)	< 0.001
HCO3-(mEq/L)	23.45	(21.75–25.5)	24.6	(23.1–26.8)	0.184
Others					
Maximum CRP (mg/dL)	24.3	(11.6–28.2)	12.2	(7.1–18.8)	0.012
Prone position* (%)	5	(41.7)	13	(22.0)	0.154
Bimodal inflammation* (%)	7	(58.3)	25	(42.4)	0.311
Tracheostomy* (%)	3	(25.0)	15	(25.4)	0.975

*BW* body weight, *CT* computed tomography, *PEEP* positive end expiratory pressure, *CRP* C-reactive protein, *BNP* brain natriuretic peptide, *4C* coronavirus clinical characterisation consortium, *APACHE* acute physiology and chronic health evaluation, *SOFA* sequential organ failure assessment, *SAPS* simplified acute physiology score.

*Frequency (%); Rest of data are expressed as medians (IQR).

### CT features and disease scoring

**Figure 2:**
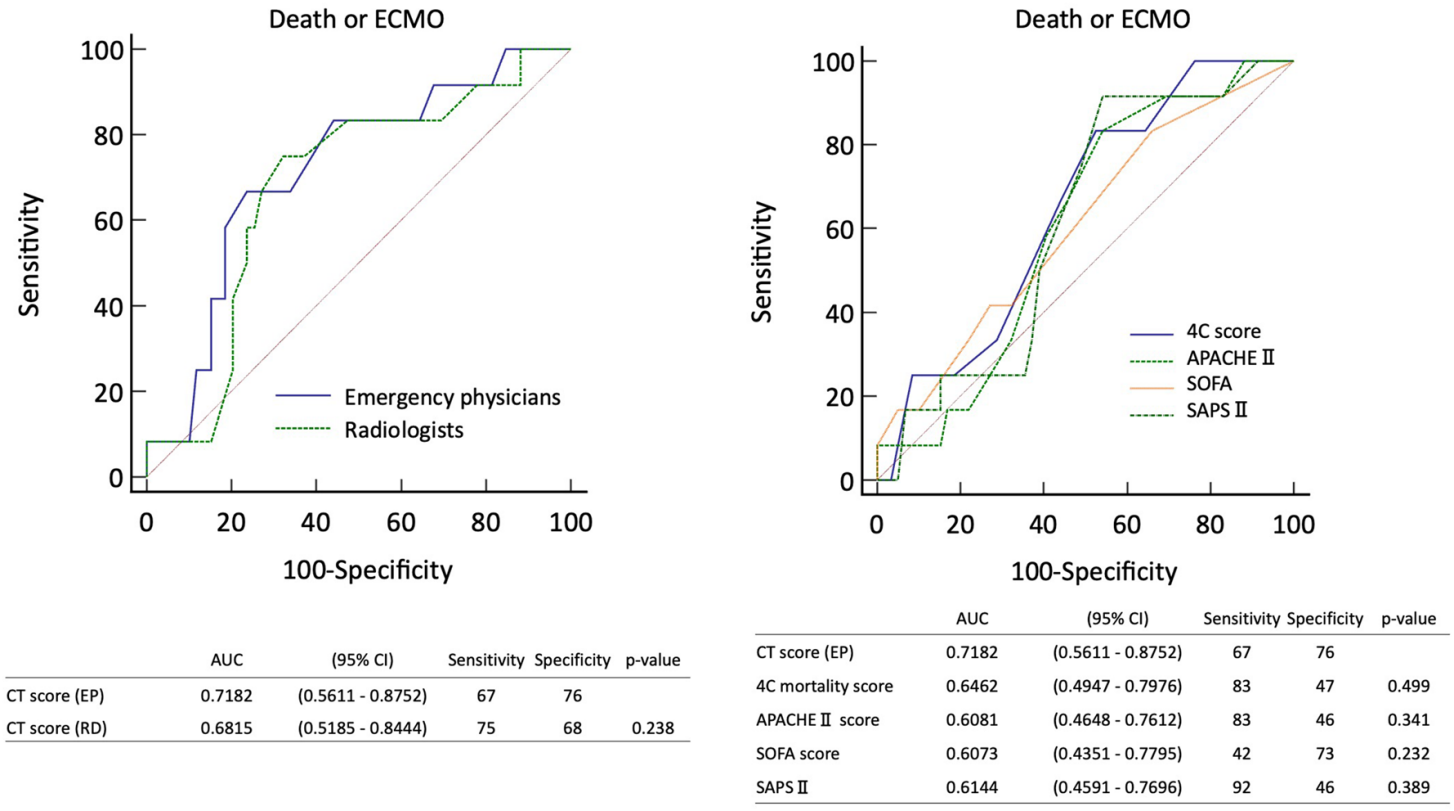
Performance of CT score and prognostic score for predicting death or ECMO management. The figure on the left shows the ROC curve for the emergency physicians versus radiologists’ results. The figure on the right shows the ROC curve for the 4C mortality score, APACHE II score, SOFA score, and SAPS II versus the CT score of emergency physicians. AUC, area under the curve; CI, confidence interval; EP, emergency physician; RD, radiologist; 4C, Coronavirus Clinical Characterisation Consortium; APACHE, Acute Physiology and Chronic Health Evaluation; SOFA, Sequential Ogan Failure Assessment; SAPS, Simplified Acute Physiology Score.

CT score was calculated as readings by two emergency physicians (mean of each score) and two radiologists (mean of each score). Of the 71 cases included in the study, 12 (16.9%) were in the death or ECMO management group, and the CT score of the emergency physicians were 17.75 and 13 for the death or ECMO group and the survival group, and 21.7 and 18 for the radiologists, respectively. The prediction of death or ECMO group by emergency physicians’ CT score was ROC of 0.718 (0.561–0.875) with a cut-off value of 16.75, sensitivity of 67%, and specificity of 76%. The death or ECMO versus survival group (median [quartiles]) had a CT score of 17.75 (14.75–20) versus 13 (11–16.5), *p* = 0.017. The radiologist CT score predicted death or ECMO group with ROC of 0.681 (0.518–0.844) with a cutoff value of 19.75, sensitivity of 75%, and specificity of 68%. The death or ECMO versus survival group (median [quartiles]) had a CT score of 21.7 (19.5–22.7) versus 18 (15–21), *p* = 0.048. The prediction of death or ECMO group by the emergency physicians and radiologists was ROC 0.718 (0.561–0.875) versus 0.681 (0.518–0.844), *p* = 0.238, with no difference between the two groups. For the prognostic score, 4C mortality score predicted death or ECMO with an ROC of 0.646 (0.494–0.797) and cutoff value of 10.5, sensitivity of 83%, and specificity of 47%; APACHE II score predicted death or ECMO with an ROC of 0.608 (0.454–0.761) and cutoff value of 9.5, sensitivity of 83%, and specificity of 46%; SOFA score predicted death or ECMO with an ROC of 0.607 (0.435–0.779) and cutoff value of 5.5, sensitivity of 42%, and specificity of 73%; and SAPS II predicted death or ECMO with an ROC of 0.614 (0.459–0.769) and cutoff value of 30, sensitivity of 92%, and specificity of 46% (Fig. 2).

CT image characteristics such as GGO, crazy paving pattern, and consolidation were read for the death or ECMO group and the survival group; GGO was 100 versus 100%, crazy paving pattern was 91.7 versus 78% (*p* = 0.040), and consolidation was 100 versus 72.9% (*p* = 0.277) between the two groups. In each zone of the CT scores of emergency physicians, the upper right was 3 versus 2 (*p* = 0.068), middle right 3 versus 2.5 (*p* = 0.176), lower right 3.25 versus 2.5 (*p* = 0.020), upper left 2.5 versus 1.5 (*p* = 0.205), middle left 2.25 versus 2 (*p* = 0.174), and lower left 3 versus 2.5 (*p* = 0.011), respectively. In each zone of the CT score of radiologists, the upper right was 3.75 versus 3 (*p* = 0.050), middle right 4 versus 3 (*p* = 0.108); lower right 4 versus 3 (*p* = 0.136); upper left 2 versus 2 (*p* = 0.759); middle left 3.75 versus 3 (*p* = 0.062); and lower left 4 versus 3 (*p* = 0.123) (Table 2).

**Table 2:** Frequency of involvement of each section with related CT score and main patterns.

	Death or ECMO		Survival group		*p*-value
	(n = 12)		(n = 59)		
CT main pattern					
GGO* (%)	12	(100)	59	(100)	–
Consolidation* (%)	11	(91.7)	46	(78.0)	0.277
Crazy paving pattern* (%)	12	(100)	43	(72.9)	0.040
X-ray					
Bilateral consolidation* (%)	4	(33.3)	20	(33.9)	0.998
Unilateral consolidation* (%)	5	(41.6)	24	(40.6)	0.998
No consolidation* (%)	3	(25)	15	(25.4)	0.998
Emergency physicians					
CT score	17.75	(14.75–20)	13	(11–16.5)	0.017
CT score > 16.7* (%)	8	(66.7)	14	(23.7)	0.003
Right upper	3	(2.5–3.5)	2	(1.5–3)	0.068
Right middle	3	(2.25–3.25)	2.5	(2–3)	0.176
Right lower	3.25	(2.75–4)	2.5	(2–3)	0.020
Left upper	2.5	(1.25–3)	1.5	(1–2.5)	0.205
Left middle	2.25	(2–3)	2	(1.5–2.5)	0.174
Left lower	3	(3–4.25)	2.5	(2–3)	0.011
Radiologists					
CT score	21.7	(19.5–22.7)	18	(15–21)	0.048
CT score > 19.7* (%)	9	(75)	19	(32.2)	0.006
Right upper	3.75	(3–4)	3	(2–4)	0.050
Right middle	4	(3–4)	3	(3–4)	0.108
Right lower	4	(3–4.5)	3	(3–4)	0.136
Left upper	2	(1.5–3.5)	2	(1–3)	0.759
Left middle	3.75	(3–4)	3	(2–4)	0.062
Left lower	4	(3–4.75)	3	(3–4)	0.123

*Frequency (%); Rest of data are presented as medians (IQR).

*CT* computed tomography, *GGO* ground-glass opacity.

The chest X-rays of patients with severe COVID-19 were also checked for pneumonia with consolidation, and it was found that 33% had bilateral consolidation, 41% had unilateral consolidation, and 25% had no consolidation. We obtained good or excellent consistency in all agreement evaluations (Table 3).

**Table 3:** Agreement in scores between paired evaluators.

Cronbach's Alpha	EP versus RD for all pts	EP1 versus EP2 for all pts	RD1 versus RD2 for all pts	EP1 versus EP2 for pts. with mild to moderate COVID
CT score	0.95	0.91	0.99	0.95
Right upper	0.91	0.87	0.98	0.92
Right middle	0.90	0.78	0.98	0.89
Right lower	0.90	0.87	1.00	0.91
Left upper	0.93	0.88	0.98	0.90
Left middle	0.91	0.83	0.98	0.90
Left lower	0.84	0.81	0.99	0.88

*EP* emergency physicians, *RD* radiologists, *pts* patients.

Multivariate analysis on death or ECMO revealed that the odds ratio (95% CI) for CT score 16.7 or higher was 8.762 (1.114–68.865), *p* = 0.039. For airway pressure 26.5 cmH_2_O or higher at intubation, odds ratio was 21.460 (1.627–282.957), *p* = 0.020. The odds ratio for maximum CRP was 1.125 (1.020–1.241), *p* = 0.018 (Table 4).

**Table 4:** Logistic analysis of clinical and CT features for COVID-19 pneumonia for death and ECMO.

Parameter	Odds ratio	95% confidence intervals	*p*-value
CT score > 16.7	8.762	(1.114–68.865)	0.039
Airway pressure at intubation > 26.5cmH_2_O	21.460	(1.627–282.957)	0.020
Maximum CRP	1.125	(1.020–1.241)	0.018
4C mortality score	1.076	(0.656–1.764)	0.770
Age	1.137	(0.956–1.353)	0.145
BW	1.051	(0.980–1.120)	0.161
Sex (men)	0.169	(0.070–4.077)	0.274

CT, computed tomography; CRP, C-reactive protein; BW, body weight; COVID-19, coronavirus disease 2019.

### CT score including moderate COVID 19 pneumonia

We evaluated the CT scores including those of patients who were hospitalized with mild to moderate COVID pneumonia without tracheal intubation during the period of the current study and who underwent CT on admission.

**Figure 3:**
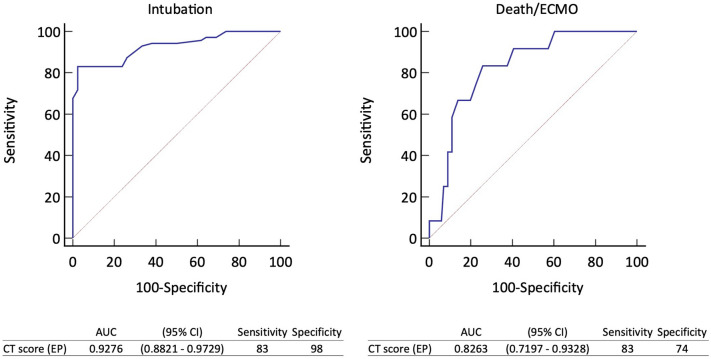
Performance of CT score for predicting intubation and death or ECMO management. The ROC curve shows the emergency physicians’ CT score for predicting intubation and death or ECMO management. AUC, area under the curve; CI, confidence interval; EP, emergency physician.

Of the 42 cases who were classified to have moderate COVID pneumonia in the study, 42 (37.5%) were in the not intubation group, and the CT score of the emergency physicians were 5.75 and 14.5 for the not intubation group and intubation group, respectively. The prediction of intubation by emergency physicians’ CT score had an ROC of 0.927 (0.882–0.972) with a cut-off value of 10.25, sensitivity of 83%, and specificity of 98%. The not intubation group versus intubation group (median [quartiles]) had a CT score of 5.75 (2–8.5) versus 14.5 (11.5–17.5), *p* < 0.0001. The prediction of death or ECMO group by emergency physicians’ CT scores had an ROC of 0.826 (0.719–0.932) with a cut-off value of 14.25, sensitivity of 83%, and specificity of 74% (Fig. 3; Table 5).

**Table 5:** Intubation, frequency of involvement of each section with related CT score, and level of agreement.

	Not intubation group		Intubation group		*p*-value
	(n = 42)		(n = 71)		
Emergency physicians					
CT score	5.75	(2–8.5)	14.5	(11.5–17.5)	< 0.0001
CT score > 14.25* (%) as cutoff for death/ECMO	0	(0)	36	(50.7)	< 0.0001
CT score > 10.25* (%) as cutoff for intubation	1	(2.3)	59	(83.1)	< 0.0001
Right upper	0.75	(0–1)	2.5	(1.5–3.5)	< 0.0001
Right middle	1	(0–1.5)	2.5	(2–3)	< 0.0001
Right lower	1	(0.5–2)	2.5	(2–3)	< 0.0001
Left upper	0.5	(0–1)	1.5	(1–2.5)	< 0.0001
Left middle	1	(0–1)	2	(1.5–3)	< 0.0001
Left lower	1	(0.5–1.5)	2.5	(2–3.5)	< 0.0001

*Frequency (%); Rest of data are presented as medians (IQR).

## Discussion

COVID-19 is not likely to be severe, but severe cases of pneumonia often lead to death^2^. Because CT is very useful in determining the severity of the disease, various scoring methods using CT have been used, and it has been found that higher scores are related to the severity of the disease^7,8,11–18^. Three key points influence the scoring by CT. The first is who reads the CTs. Reports on CT score often indicate that radiologists read the CTs. In most cases, radiologists with approximately 10 years of experience were selected, but in many cases, there were at least two radiologists with 3–18 years of experience^12,13,19–21^. The second is how to determine the region of the lung to be scored. Most often, the lungs are read in five lobes (three right and two left lobes) along the anatomical region^7–9,11–16^. A different way to read the lungs is to divide them into three zones: upper, middle, and lower. The upper section is from the apex of the lung to the bronchial bifurcation, the middle section is from the bronchial bifurcation to the right inferior pulmonary vein, and the lower section is from the right inferior pulmonary vein to the diaphragm, for a total of six locations on both sides^17,18^. In addition to this division, there is another method in which the lungs are divided into anterior and posterior sections for a total of 12 regions for reading^19^. The third is the method of scoring the divided regions. Most of the time, each area is scored out of 5 points: 0, no pneumonia; 1, 1–5% pneumonia; 2, 6–25% pneumonia; 3, 26–50% pneumonia; 4, 51–75% pneumonia; and 5, > 75% pneumonia^7,8,11–13,15^. A 4-point scale not separated by 5% (0, 0%; 1, 1–25%; 2, 26–50%, 3, 51–75%; 4, > 75%)^9,14,19^, is more convenient than a 2-point scale (0, 0%; 1, 1–50%; 2, > 50%)^20,21^.

These three points have both advantages and disadvantages. Regarding the first point, it is definitely advantageous to be sure that radiologists will be reading the data. However, the disadvantage is that they are not necessarily stationed at the hospital where the COVID patients are admitted. If the scoring is not available to the physician who will examine the patient and read the CT at that time, the advantage is lowered. Regarding the second point, the area of the lungs, there are many methods that read the lungs in five lobes, but this reading has the disadvantage of not equally dividing the left and right sides of the lungs. In general, the right lung is divided into three lobes (upper, middle, and lower lobes), and the left lung into two lobes (upper and lower lobes), each of which is not equal in size^22^. Therefore, scoring each lobe equally by a factor of 5 may not represent the extent to which the lungs are actually affected by pneumonia. Finally, regarding the third point, it is good to score the absence of pneumonia as zero; however, it is difficult to determine the range within which the points should be separated. If one believes that a patient with pneumonia is more likely to have a mild case if the range is small, one might divide the points between 1 and 5%, or if one believes that a patient with pneumonia in the range of less than 25% is not different from a patient with pneumonia in the range of less than 5%, one might not divide the points between 5% and 1–25%, since fewer points are better. The scoring might be 1–25%, instead of 5%. Although advantages and disadvantages exist for each point, all scoring systems associate higher scores with a worse prognosis.

All our patients had severe COVID-19 pneumonia and were intubated and required ventilator management. We used a 3-zone technique (six zones in total) with a score distribution of 0–5, so that the left and right sides were equally divided^23^. Among severely ill patients, a higher CT score was more likely to result in death or ECMO introduction. CT images showed a higher rate of crazy paving pattern and consolidation in the death and ECMO groups. On multivariate analysis, our results showed that patients with airway pressure higher than 26.5 cmH_2_O and CT score higher than 16.7 were significantly more likely to die or receive ECMO.

This suggests that the CT score is useful in predicting the prognosis of patients with COVID-19 pneumonia, but not every facility has radiologists. Therefore, it is difficult for physicians, who are not radiologists, to assign CT scores. Emergency physicians, who often see COVID-19 pneumonia in the initial treatment, were asked to read the case and compare their prognostic predictions with those of radiologists. There was no statistical difference in prognostic prediction between emergency medicine specialists and radiologists with ROC 0.718 (0.561–0.875) versus 0.681 (0.518–0.844), *p* = 0.238. This suggests that even emergency physicians may find it useful to use CT score as a prognostic predictor. However, a comparison of the two scores showed that the radiologists scored the CT images higher than the emergency physicians (Table 2). This could be due to the difference in the reading of the GGO between normal lungs and the lungs with pneumonia. If crazy paving pattern or consolidation was present, it was easy to assume that there was pneumonia in that area. The reason for the higher score given by the radiologists was thought to be the difference in the reading of the frosted shadows. The emergency physicians did not consider a small amount of frosted shadows to be pneumonia, whereas the radiologists did, which may have contributed to the higher scores. The presence or absence of a crazy paving pattern or consolidation affects the severity of the disease, which can be read by emergency physicians; therefore, there was no difference in prognosis between emergency physicians and radiologists.

We included cases of mild to moderate COVID-19 because the CT score can also predict whether intubation is necessary. The scores varied significantly that even emergency physicians could easily determine the need for intubation. However, in this study, it was found to be useful specifically in determining whether ECMO is needed in severe COVID cases. This is because severe COVID pneumonia has various presentations, which makes it difficult to determine the severity of the disease. In addition, there are ICUs where ventilator management is available, but ECMO is not. In such cases, there is the advantage of a faster decision to transfer the patient to a facility where ECMO can be used.

However, not every facility is staffed by radiologists and is equipped to introduce ECMO 24 h a day. COVID is a pandemic, and it is not always possible to treat patients in hospitals equipped with such facilities. A complementary method is a deep-learning reading system, the usefulness of which has been previously reported^24^. We conducted this study in the hopes that our generated CT score could be a tool that could help in situations where it was not possible to prepare such a score. The present results suggest that the CT scoring of COVID patients is important and useful for determining future treatment strategies.

### Limitations

This study has several limitations. First, the use of selected representative axial CT images may not allow an accurate assessment of pulmonary opacity if the distribution of lesions in each lung lobe is unbalanced. Second, because we were responsible for severe COVID, not all cases were evaluated by CT under the same conditions, as CT may have been performed at the transfer site. Third, this study was conducted only in severely ill patients and not in moderately ill patients. Fourth, it is important to note that long-term mortality and post-discharge prognostic symptoms were not considered. Further studies are needed to determine whether the CT score derived from representative CT images can predict the long-term prognosis of patients after discharge.

## Conclusions

A higher score on our generated CT score could predict the likelihood of death or ECMO management. The CT score also showed no difference in the predictive ability between radiologists and emergency physicians, suggesting that it can be used by non-radiologists. A CT score at the time of admission allows for early preparation and transfer to a hospital that can manage patients who need ECMO.

## Supplementary Information

Supplementary Information 1.

Supplementary Information 2.

Supplementary Information 3.

## Data Availability

The dataset supporting the conclusions of this study is included within the article (and its supplementary information files).

## Abbreviations

WHO: World Health organization
COVID-19: Coronavirus disease 2019
RT-PCR: Reverse transcriptase-polymerase chain reaction
CT: Computed tomography
GGO: Ground-glass opacity
ECMO: Extracorporeal membrane oxygenation
CRP: C-reactive protein
BNP: Brain natriuretic peptide
ABG: Arterial blood gas
PEEP: Positive end-expiratory pressure
P/F: PaO_2_/F_I_O_2_
VV-ECMO: Venovenous extracorporeal membrane oxygenation
4C: Coronavirus clinical characterisation consortium
APACHE: Acute physiology and chronic health evaluation
SOFA: Sequential organ failure assessment
SAPS: Simplified acute physiology score
