# Supplementary material for: Prediction of prognosis in patients with severe COVID-19 pneumonia using CT score by emergency physicians: a single-center retrospective study
Source: Sci Rep. 2023 Mar 10;13:4045. doi: 10.1038/s41598-023-31312-5 (PMC10004443; doi:10.1038/s41598-023-31312-5)
Supplement: Supplementary file 3 — Supplementary Information 3. [file 41598_2023_31312_MOESM3_ESM.docx]

**Prediction of prognosis in patients with severe COVID-19 pneumonia using CT score by emergency physicians: A single-center retrospective study**

Yasufumi Oi^1,2*^, Fumihiro Ogawa^1,2^, Tsuneo Yamashiro^3^, Shoichiro Matsushita^3^, Ayako Oguri^1,2^, Shusuke Utada^1,2^, Naho Misawa^1,2^, Hiroshi Honzawa^1,2^, Takeru Abe^2,4^, Ichiro Takeuchi^1,2,4^

^1^Emergency Care Department, Yokohama City University Hospital, Yokohama, Japan

^2^Department of Emergency Medicine, Yokohama City University School of Medicine, Yokohama, Japan

^3^Department of Radiology, Yokohama City University School of Medicine, Yokohama, Japan

^4^Advanced Critical Care and Emergency Center, Yokohama City University Medical Center, Yokohama, Japan

**Supplementary Information**

File name: Supplementary Material 1

File format: xlsx

Title of data: CT score data from severe COVID-19 patients

Description of data: CT score data from severe COVID-19 patients

File name: Supplementary Material 2

File format: xlsx

Title of data: CT score data from mild to moderate COVID-19 patients

Description of data: CT score data from mild to moderate COVID-19 patients
